# Attentional avoidance in peer victimized individuals with and without psychiatric disorders

**DOI:** 10.1186/s40359-019-0284-1

**Published:** 2019-02-22

**Authors:** Benjamin Iffland, Angelina Weitkämper, Nicolai J. Weitkämper, Frank Neuner

**Affiliations:** Department of Psychology, Bielefeld University, Postbox 100131, 33501 Bielefeld, Germany

**Keywords:** Child maltreatment, Peer victimization, Attention, Attentional bias, Attentional avoidance

## Abstract

**Background:**

Attentional biases are a relatively robust phenomenon among clinical populations but less pronounced in healthy participants. However, regarding the components of attentional biases and the directions of attention allocation, there are several inconsistencies in the literature. The present study examined whether these inconsistencies can be traced back to previous experiences of relational peer victimization in clinical populations.

**Methods:**

Participants were subjects with a diagnosed psychiatric disorder (*n* = 30) and healthy controls (*n* = 31). Additionally, the sample was divided into two subgroups according to the participants’ reports of previous relational peer victimization (high peer victimization: *n* = 28; low peer victimization: *n* = 33). Attentional biases were measured by the Emotional Stroop task and a dot-probe task.

**Results:**

In both samples, peer victimized participants showed delayed response times when color-naming negative and positive compared to neutral adjectives in the Emotional Stroop task. Likewise, the dot-probe task indicated attentional avoidance of both negative and positive words in peer victimized participants with and without a psychiatric disorder. Interestingly, presence of a psychiatric disorder did not have a significant effect on attentional biases.

**Conclusion:**

Both tasks could detect that attentional processes were linked to the experience of peer victimization rather than to the current diagnostic status of the participants. Attentional avoidance of emotional stimuli may prevent victimized individuals from responding adequately to environmental stimuli, which may increase the risk for the development of psychopathology.

## Background

A large body of research has demonstrated that attentional biases are a relatively robust phenomenon among anxious populations, but less pronounced and consistent in non-anxious subjects [1–5]. Generally, attentional biases lead individuals to selectively and differentially allocate attention towards threatening stimuli in comparison to neutral stimuli. In particular, attentional biases characterized in research can be divided into facilitated attention (i.e., faster detection of threat vs. non-threat stimuli), difficulty in disengagement (i.e., disengaging attention from threat stimuli is harder than from a neutral stimulus), and attentional avoidance (i.e., shifting attention towards locations opposite the location of threat; for a review, see [1]).

The most commonly used task to measure attentional bias is the modified or Emotional Stroop task [6]. In this task, different types of words (e.g., threatening and neutral) are displayed in varying colors. Subjects are asked to name the colors while ignoring the semantic contents of the words. Slower response times to color-naming of threat words compared to neutral words are considered an indication of an attentional bias. However, interpretation of the attentional bias measured by the Emotional Stroop task is difficult. That is, delayed response times to threat words may be due to enhanced attention towards threat as well as a general delayed responding to threat [7]. Furthermore, these aspects of difficulty in disengaging and facilitated attention (also referred to as vigilance or attentional orienting) are not addressed in the Stroop task. The dot probe task [8] was established and improved [9] in order to resolve this problem, disentangling the different components of attentional biases. In this task, two words appear on a computer screen with one word above or beside the other for a brief duration. Then, a probe appears in the location of one of the two words. Subjects indicate which stimulus the probe replaced. Different response times towards probes that replace threatening compared to neutral stimuli indicate the presence of attentional biases. Here, difficulty in disengagement is present when subjects are slower in indicating the probe when it appears in a different location than the threat word, whereas an attentional orienting is present when subjects are faster in indicating the probe when it appears in the location of the threat word [9].

From a clinical point of view, attentional biases are of interest because they are most likely relevant in the development and maintenance of psychiatric disorders [10, 11]. For instance, the schema-based model of information-processing by Beck and Clark proposed that anxiety disorders are caused by different cognitive processes (e.g., [12, 13]). According to this model, cognitive biases in information processing are reflected by selective attention to threat, interpretation of ambiguous stimuli as threatening, selective recall of threatening experiences, and an expectancy of aversive events [14, 15]. That is, it is suggested that attentional biases influence individuals’ everyday lifes and interactions by influencing, for instance, if threatening cues (e.g., angry faces) are detected in a room, if a peer’s comment is interpreted as a negative evaluation, in which way a student evaluates and recalls his performance in a presentation, and if a danger or reward is expected in the next encounter with a peer. In line with this assumption, attentional biases could be detected in several studies examining subjects with high trait anxiety and clinical anxiety [2]. Using the Emotional Stroop task, attentional biases have been found in patients with posttraumatic stress disorder (PTSD) [16, 17], panic disorder [18], generalized anxiety disorder (GAD) [19], obsessive-compulsive disorder [20], social phobia [21–23], and specific phobia [24]. In these studies, patients with anxiety disorders showed increased reaction times towards disorder-relevant words when compared to neutral or positive words. Similarly, attentional biases have been demonstrated in the dot-probe task for patients with GAD [25], social phobia [26–28], and PTSD [29, 30].

Although the majority of studies demonstrated a difficulty in disengagement among anxious individuals [1, 9, 31, 32], there are also studies presenting converse or even no bias effects. For example, attentional avoidance, rather than disengagement, was found in socially anxious subjects [27, 28]. Moreover, there are several studies failing to show any attentional bias in PTSD patients towards trauma-related pictures, threatening faces or other disorder-related stimuli [33–35]. Additionally, studies applying the dot-probe task showed null results in patients with panic disorders [36], and obsessive-compulsive disorder (OCD) [37–39]. Likewise, the literature of Emotional Stroop studies presents inconsistent results. For example, [40] did not find any differences in reaction times towards threat words between patients with panic disorder or OCD and healthy controls. Similarly, Moritz and colleagues (2004) could not show interference effects in patients with OCD [41].

With respect to attentional biases in other psychiatric disorders, the current body of literature is peppered with inconsistencies as well. A meta-analysis of 29 empirical studies examining depressive patients demonstrated an attentional bias towards negative information in this population [42]. While depressive patients differed significantly from controls in the dot-probe task, there was only a marginal difference between depressive subjects and healthy controls in the Emotional Stroop task. Other studies could not find any differences between depressive and healthy subjects using the Emotional Stroop [43] or the dot-probe task [44]. In patients with personality disorders, particularly borderline personality disorder, facilitated attention towards negative emotional stimuli was found in an Emotional Stroop task [45]. However, von Ceumern-Lindenstjerna and colleagues (2010) could determine that an attentional bias towards negative faces was not due to Borderline personality disorder per-se but to an interaction between mood and the personality disorder, i.e., only patients with negative mood showed facilitated attention towards threatening stimuli [46]. Furthermore, attentional biases were demonstrated in patients with schizophrenia and psychotic disorders [47–49]. Again, attention was allocated towards disorder-related stimuli.

Accordingly, it appears that trait anxiety and psychopathology are associated with attentional biases. However, this association seems to be moderated by both parameters within the paradigms used to detect biases (i.e., threat intensity and stimulus duration) [1] and individual differences that lie beyond trait anxiety and psychopathology. The former suggestion is built upon findings that attentional biases in high trait anxious individuals were more easily found when highly, but not mildly, threatening stimuli were presented, and that facilitated attention was associated with a rather quick presentation of stimuli [1]. The latter assumption is supported by studies reporting attentional biases in healthy subjects, as well [9, 50]. Adverse child experiences (ACEs) are a potential candidate in distorting attentional processes and moderating the association between psychopathology and attentional biases. Consider that ACEs during childhood and adolescence have lasting consequences and contribute to different psychological disorders including depression and anxiety disorders [51–55]. Indeed, recent studies have shown that attentional biases were present in patients with a history of ACEs but not in patients who did not report ACEs in their childhood and adolescence [56, 57]. For example, Günther, Dannlowski, Kersting, and Suslow (2015) reported that in a sample of depressive patients facilitated attention towards sad faces was heightened in subjects reporting ACEs [58]. Furthermore, attentional biases towards negative stimuli were also reported in healthy subjects with ACEs [59–61].

To date, most studies examined ACEs including low maternal care, parental conflicts [62, 63], or physical or sexual transgression by caretakers [64–68]. However, there are also social experiences that involve emotional abuse and neglect by caretakers [69, 70] as well as emotional forms of abuse by peers. The latter are also referred to as relational peer victimization and are characterized by bullying, verbal threats or aggression, malicious manipulation of a relationship, friendship withdrawal, and damaging another’s peer relationships [71]. Recently, it has been proposed that emotional types of maltreatment lead to psychological consequences that can be as severe as the outcomes of physical or sexual maltreatment [70, 72]. In particular, experiences of relational peer victimization increase the risk of various forms of psychopathology [73]. For instance, peer victimization is linked to sub-clinical as well as clinical social anxiety disorder (SAD) [71, 74–81]. Accordingly, Rosen, Milich, and Harris (2007) proposed a modified social-information-processing model in which the activation of a so-called victim schema initiates hypervigilance for threatening cues and an attentional bias to threatening compared to non-threatening cues in social interactions [82]. In line with this assumption, children who reported more frequent experiences of victimization responded more quickly to victim-related words in an Emotional Stroop task [82]. To our knowledge, however, there are no studies examining the extent to which experiences of relational peer victimization contribute to the implementation of attentional biases in adults with and without psychiatric disorders.

The aim of the present study was to address inconsistencies in the existing literature about attentional biases in clinical samples. Here, a large body of studies presenting an attentional bias towards negative stimuli [16, 30, 46] conflicts with recurrent reports of either converse or null effects [27, 33, 34, 40, 43]. Recent studies reporting effects of ACEs on attentional processes in clinical as well as healthy samples suggest that negative life experiences may serve as a moderator of the magnitude of attentional biases [56, 58–60]. Here, given its effect on a wide range of psychopathology, the contribution of peer victimization to the development of attentional biases seems to be underrepresented in the literature.

The current study sought to address this underrepresentation by examining the influence of peer victimization on attentional biases as measured by the Emotional Stroop task and the dot-probe task in two samples consisting of subjects with either a diagnosed psychiatric disorder or healthy controls. With respect to the existing literature, we hypothesized that subjects with a diagnosed psychiatric disorder would show an attentional bias towards negative compared to neutral adjectives. Furthermore, we assumed that the attentional bias would be more pronounced in subjects with a history of peer victimization irrespective of their current diagnostic status. When comparing positive to neutral adjectives, we did not expect to find any attentional biases.

## Method

### Participants

Due to the study’s aims, recruitment of participants was two-pronged. The clinical sample was recruited through the Hans-Peter-Kitzig-Institut (Gütersloh, Germany), a regional rehabilitation hospital for patients with psychiatric disorders. The healthy control sample was recruited through online advertisements in student newsgroups and bulletins at the campus of Bielefeld University. Advertisements informed that the study examined the association of personality traits, life experiences, and attentional processes.

The total sample consisted of 61 participants, (26 females, 42.6%). Out of the whole sample, 30 individuals (49.2%) represented the clinical sample. Exclusion criteria for the clinical sample included (a) evidence of a current substance abuse or dependence, (b) evidence of current active-phase symptoms of psychosis as delusions, and hallucinations, and (c) evidence of acute suicide intention or ideation. Number and types of diagnoses of the clinical sample are presented in Table 1. For the healthy control group, 32 individuals were screened for participation initially. One individual was excluded because criteria for a current mental disorder were fulfilled. Accordingly, the 31 individuals (50,8%) comprising the control sample reported no current mental or neurological disorders, no current use of prescriptive medication except oral contraceptives, and no current alcohol or drug dependence. Out of the control sample, 30 individuals were students at university and one reported to be working full time. Eligible participants of both groups read and signed an informed consent form that was approved by the Ethics Committee of Bielefeld University. Participants of the healthy control sample either received course credit or a compensation for their time of 6€/hour. The demographic characteristics of the two groups and diagnoses of the clinical sample are presented in Table 1.

**Table 1:** Participant characteristics and mean values on the assessments (*N* = 61)

	Cronbach’s *α*	Total (*N* = 61)	Psychiatric patients (*n* = 30)	Healthy controls (*n* = 31)	*p*
Age, *M* (*SD, range*)		24.59 (4.97, 18–40)	26.20 (6.12, 18–40)	23.03 (2.83, 19–33)	.014^e^*
Gender, *% female* (*n*)		62.5 (30)	40.0 (12)	45.2 (14)	.797^f^
Family status, *% single* (*n*)		52.1 (25)	83.3 (25)	58.1 (18)	.049^f^*
Peer Victimization^a^, *M* (*SD*)	.91	12.02 (8.21)	16.50 (8.66)	7.68 (4.78)	< .001^e^***
Symptoms of Depression^b^, *M* (*SD*)	.91	12.41 (9.77)	19.33 (9.25)	5.71 (3.73)	< .001^e^***
General Psychopathology^c^, *M* (*SD*)	.98	.72 (.70)	1.15 (.72)	.29 (.33)	< .001^e^***
Trait Anxiety^d^, *M* (*SD*)	.95	46.72 (5.65)	47.62 (5.50)	45.82 (5.74)	.220^e^
Childhood Trauma Questionnaire, *M* (*SD*)	.93	53.80 (5.32)	54.23 (6.74)	53.39 (3.52)	.539^e^
Emotional Abuse, *M* (*SD*)	.86	10.10 (4.75)	12.51 (5.06)	7.77 (3.01)	< .001^e^***
Emotional Neglect, *M* (*SD*)	.86	10.43 (4.32)	12.59 (4.20)	8.42 (3.40)	< .001^e^***
Physical Abuse, *M* (*SD*)	.84	6.30 (2.81)	6.90 (3.69)	5.71 (1.40)	.106^e^
Sexual Abuse, *M* (*SD*)	.94	5.66 (2.35)	5.85 (2.62)	5.48 (2.08)	.547^e^
Principal diagnoses					
Major depressive disorder, single episode, *%* (*n*)			16.6 (5)		
Major depressive disorder, recurrent, *%* (*n*)			40.0 (12)		
Bipolar disorder, *%* (*n*)			3.3 (1)		
Social phobia, *%* (*n*)			6.6 (2)		
Obsessive-compulsive disorder, *%* (*n*)			3.3 (1)		
Borderline personality disorder, *%* (*n*)			13.3 (4)		
Mixed and other personality disorder, *%* (*n*)			3.3 (1)		
Paranoid schizophrenia, *%* (*n*)			6.6 (2)		
Schizoaffective disorder, *%* (*n*)			6.6 (2)		

* *p* < .05, ** *p* < .01, *** *p* < .001; ^a^Fragebogen belastender Sozialerfahrungen; ^b^Beck Depression Inventory; ^c^Brief Symptom Inventory - Global Severity Index; ^d^ State Trait Aniety Inventory-Trait; ^e^independent Student’s *t*-test, ^f^*Chi*-*squared* test

### Diagnostic status

Information about the diagnostic status (i.e., number and type of diagnoses) of the clinical sample was obtained from the participants’ health records of the Hans-Peter-Kitzig Institut. Diagnostic status of the control sample was assessed using the German Version of the Mini International Neuropsychiatric Interview (M.I.N.I.) [83–85]. The M.I.N.I. is a structured clinical interview designed to generate diagnoses for the main Diagnostic and Statistical Manual-III-R/IV Axis I disorders. The interviews were conducted by Master-level clinical psychologists who were trained in the application of the M.I.N.I.. Participants in the control sample were eligible for the study when no current or lifetime diagnosis was present.

### Materials

The stimulus set consisted of 180 adjectives (negative, neutral, positive) and was derived from prior studies on word processing [86–88]. In these studies, adjectives had been rated in terms of valence and arousal in an interpersonal evaluative context. Because peer victimization most commonly implies negative evaluations by others (overt as well as implicit), it was suggested that adjectives with a social evaluation connotation would be of special interest and suitable to detect attentional biases in the context of peer victimization. Out of these 180, 60 adjectives (the 20 most negative, the 20 most neutral, and the 20 most positive) were selected for the Emotional Stroop task and 80 adjectives (the 20 most negative, the 40 most neutral, and the 20 most positive) were selected for the dot-probe task (see Table 2). The selected adjectives were matched in their linguistic properties, such as word length and frequency within each task (see Table 3). Negative and positive adjectives differed in their valence only. With respect to previous experiences with the stimulus set, neutral adjectives were allowed to be less arousing [86–88].

**Table 2:** List of adjectives that were selected for the Emotional Stroop task and the dot-probe task

Negative adjectives	Neutral adjectives	Positive adjectives
antisocial	abstinent^a^	beautiful
artificial	accented	confident
awkward	affable^a^	courageous
brazen	ambitious	cute
cagey	angular	enamored
cold	bourgeois^a^	exciting
cold-hearted	casual^a^	funny
disgusting	chronological^a^	humorous
distressing	commonly	jokey
foolish	complaisant^a^	mature
lavish	conformed^a^	optimistic
meaningless	countless^a^	passionate
nasty	devout	seductive
pretentious	domestic	sentimental
savage	economical	smart
stupid	empirical^a^	stunning
submissive	exact	superb
ugly	frothy	tender
uninspired	heroic^a^	thrilling
unstable	innocuous^a^	vivacious
	juridical^a^	
	licensed	
	medical	
	neutral^a^	
	northwest^a^	
	numerous	
	official^a^	
	principally^a^	
	provisional	
	prudential	
	regularly	
	resting^a^	
	right-handed^a^	
	sentimental	
	statutorily	
	streaked	
	subjective^a^	
	symmetric^a^	
	tame	
	unconscious	

^a^ neutral adjectives were used in both tasks; neutral adjectives not indexed were used in the dot-probe task only

**Table 3:** Comparisons of negative, neutral, and positive adjectives by one-way-analyses of variances

	Negative adjectives	Neutral adjectives	Positive adjectives	*F*
Emotional Stroop task				*df* (2,57)
Valence, *M* (*SD*)	2.16^a^ (.47)	4.89^b^ (.15)	7.98^c^ (.62)	819.04***
Arousal, *M* (*SD*)	5.58^a^ (.68)	3.12^b^ (.93)	5.33^a^ (.79)	56.43***
Word length, *M* (*SD*)	8.70 (3.03)	9.10 (2.05)	9.30 (3.03)	.25
Word frequency (per million), *M* (*SD*)	2.60 (3.62)	5.28 (7.73)	5.26 (4.96)	.24
Dot-probe task				*df* (2,77)
Valence, *M* (*SD*)	2.16^a^ (.47)	4.92^b^ (.28)	7.98^c^ (.62)	902.83***
Arousal, *M* (*SD*)	5.58^a^ (.68)	3.14^b^ (.81)	5.33^a^ (.79)	89.90***
Word length, *M* (*SD*)	8.70 (3.03)	9.00 (2.41)	9.30 (3.03)	.24
Word frequency (per million), *M* (*SD*)	2.60 (3.62)	6.63 (9.32)	5.26 (4.96)	2.02

***p ≤ .001. Means in the same row sharing the same superscript letter do not differ significantly from one another at p ≤ .05 based on LSD test post hoc comparisons; data on word frequency are based on the CELEX database

### Paradigms

#### Emotional Stroop task

The Emotional Stroop task consisted of 240 trials. In total, 80 negative, 80 neutral, and 80 positive words were shown, in each case 20 words were colored in red, 20 in blue, 20 in green, and 20 were colored in yellow on a black background. Each single word was presented four times. Stimuli were shown throughout until the participants responded. After an intertrial interval of 200 ms the next stimulus was presented. Participants’ task was to identify the color of the presented words as quickly and as accurately as possible. Participants indicated their response by pressing buttons on a keyboard with the index and middle fingers of both hands. In order to reassure that the participants were able to assign the colors to the right buttons, the assignment of buttons and colors was presented on the screen throughout the experiment. The assignment of buttons was counterbalanced across participants. The order of words, word valences, and colors was randomised. We used the software package Inquisit 4.0.3 (Millisecond Software, Seattle, WA, USA) to deliver stimuli and record responses and reaction times (RTs).

#### Dot-probe task

The dot-probe task consisted of two blocks of 240 trials each, with a short break between the blocks. There were three different types of trials in the present task: negative–neutral, positive-neutral, and neutral-neutral, with negative and neutral, neutral and neutral, and positive and neutral words combined, respectively. All words were presented in black on a white background, lowercase. The word pairs were presented with one word beside the other (horizontal) in the middle of the screen. The dot-probe experiment began with 12 practice trials using neutral–neutral word pairs to familiarize participants with the task. Each trial started with a black fixation cross presented in the center of a white screen for 500 ms. Then, a word pair appeared with one word beside the other for 500 ms. A gray dot emerged in one of the word locations immediately after the offset of the words. The location of the target word (left or right) and probe (left or right) was randomized for all trials. The inter-trial interval for all trials was 500 ms. Participants were instructed to respond as quickly and as accurately as possible and to indicate the location of the gray dot (left or right) by pressing either the button “E” (left) or “I” (right) on a keyboard with their index fingers of both hands. The three types of word pairs were randomly formed. Each word was presented six times (3 times on each side) for a total of 480 experimental trials. The combination and order of word pairs varied randomly for each participant. We used the software package Inquisit 4.0.3 (Millisecond Software, Seattle, WA, USA) to deliver stimuli and record responses and reaction times (RTs).

### Procedure

Prior to the laboratory session, participants were asked to fill in a questionnaire assessing relational peer victimization (*Fragebogen zu belastenden Sozialerfahrungen, FBS* [Adverse Social Experiences Questionnaire]) [89]. The FBS consists of 22 items describing aversive social situations like rejection, exclusion, being laughed at, insulted, and teased by peers (e.g., “I was excluded from games or activities by other children or adolescents”, “I have been laughed at in the presence of other children”). For each situation, respondents were asked whether or not they have experienced this situation during childhood (age 6 to 12) or adolescence (age 13 to 18). The total score is calculated as a sum of “Yes” responses across both age periods and ranges from 0 to 44. The total-score of the FBS presented with a satisfying stability over a 20-month period (*r* = .89) [89]. Construct validity has been confirmed through correlations with measures of psychological symptom distress and social anxiety. Moderate correlations with the scales of the Childhood Trauma Questionnaire [90], as well as an incremental contribution to the prediction of psychopathology, support the idea that the FBS assesses an additional construct of child maltreatment [74, 89]. The FBS was applied in several studies examining the role of peer victimization in terms of psychopathology and psychophysiology before suggesting a good fitness of the instrument (e.g., [74, 81, 91–93]). Additionally, participants were asked to complete an assessment battery including a socio-demographic questionnaire as well as well-established questionnaires for child maltreatment (German version of the Childhood Trauma Questionnaire, CTQ) [90, 94], symptoms of depression (German version of the Beck Depression Inventory II, BDI-II) [95, 96], general psychopathology and psychological distress (German version of the Brief Symptom Inventory, BSI) [97–99], and trait anxiety (German version of the State Trait Anxiety Inventory-Trait) [100, 101]. In the current sample, we obtained good to excellent internal consistency on all scales (see Table 1). Once completed, participants of the control sample were administered the M.I.N.I. [84] to determine diagnostic status. Afterwards, the Emotional Stroop task and the dot-probe task were used to detect any attentional biases in participants. The presentation order of tasks was counterbalanced across participants. Instructions for the tasks were presented on the computer screen for the participants to read. After completion of the tasks, participants were debriefed.

### Data reduction and statistical analyses

In the Emotional Stroop task an attentional bias is indicated by greater color-naming latencies following negative/positive words in comparison with neutral words [5]. Therefore, difference scores for the reaction times (RT) in color-naming negative and neutral as well as positive and neutral words were calculated (Emotional Stroop Index = RT negative/positive words – RT neutral words). Positive scores indicate a greater attentional bias in the processing of negative and positive words. Consistent with procedures of prior studies, trials with reaction times lower than 300 ms or higher than 4000 ms were excluded from analyses [39, 102]. In addition, trials where participants indicated the wrong color (error trials) were excluded. Error rates did not differ between the two samples. Out of 240 trials, participants indicated between 0 and 17 wrong colors (clinical sample: *M* = 5.65, *SD* = 4.17; control sample: *M* = 6.29, *SD* = 4.26; *t* (57) = .58, *p* = .562). No participants were excluded due to higher error rates than 25%. Outliers were defined as participants presenting reaction times that deviated more than three SDs from mean reaction times and were removed from analyses. Outliers were present in both the clinical (*n* = 1) and the control sample (*n* = 1).

For the dot-probe task, attentional bias scores were calculated for two different trial types (i.e., negative-neutral and positive-neutral). Here, an attentional bias is indicated by either lower RTs to the probe if it emerges at the location where the participants were focusing their attention, or higher RTs to the probe when it appears in the location where the participants were not attending [103]. Particularly, the attentional bias scores are calculated by subtracting participants’ RTs to the probe when it appears in the same position as the target word from participants’ RTs to the probe when it does not appear in the same position as the target word [103, 104]. In the present study, the target words were the negative words in the negative–neutral trials, and the positive words in the positive–neutral trials. According to previous research [103, 104], significant positive bias scores indicate that participants were focusing their attention on the area around the target words when the probe occured, whereas significant negative bias scores indicate that participants were not attending to the area around the target words when the probe occurred.

To better understand the mechanisms underlying the attentional bias, additional index scores were calculated to differentiate vigilance and difficulty to disengage [9]. Vigilance should lead to faster responses on trials where the probe appeared where participants were attending compared to neutral trials. This would indicate that participants preferentially hold their attention at the target location. Specifically, the Orienting Index scores are calculated by subtracting participants’ RTs to the probe when it occurs in the same position as the target word from participants’ RTs to the probe when two neutral words were presented [9]. Should participants have difficulties in disengaging attention from valenced words, this would result in slower reaction times on trials where the probe appeared in a location they were not attending to due to the time needed to shift attention from the valenced to the neutral location. Specifically, the Disengaging Index scores are calculated by subtracting participants’ RTs to the probe when two neutral words were presented from participants’ RTs to the probe when it does not occur in the same position as the target word [9]. All bias and index scores must differ significantly from zero to confirm that an absolute attentional bias exists. In addition, a relative bias is indicated by significantly differing bias/index scores between two groups.

In line with previous studies, trials with reaction times lower than 150 ms or higher than 2000 ms were excluded from analyses [9, 105, 106]. In addition, trials where participants indicated the wrong location of the probe (error trials) were excluded. Error rates did not differ between the two samples. Out of 480 trials, participants indicated between 0 and 38 wrong locations (clinical sample: *M* = 10.52, *SD* = 10.12; control sample: *M* = 8.13, *SD* = 5.71; *t* (56) = 1.11, *p* = .273). No participants were excluded due to higher error rates than 25%. Outliers were defined as participants presenting reaction times that deviated more than three SDs from mean reaction times and were removed from analyses. Outliers were present for the clinical (*n* = 1) and the control sample (*n* = 2). Accordingly, the remaining sample for the analyses of the Emotional Stroop task consisted of 59 participants (30 clincial sample participants, 50.8%) and for the analyses of the dot-probe task of 58 participants (29 clinical sample participants, 50.0%).

All statistical analyses were carried out using the Statistical Package for the Social Sciences 25. Because age differed significantly between the two samples, all ANOVAs were carried out with age serving as a covariate. Initially, an omnibus 2 (group: clinical vs. control sample) × 2 (peer victimization: high vs. low) × 3 (valence: negative, neutral, positive) analysis of covariance (ANCOVA) with repeated measures on valence and age serving as a covariate was calculated for the mean RTs of the Emotional Stroop task. Similarly, an omnibus 2 (group: clinical vs. control sample) × 2 (peer victimization: high vs. low) × 2 (valence: negative vs. positive) × 2 (location of the dot: congruent vs. incongruent) analysis of covariance (ANCOVA) with repeated measures on valence and age serving as a covariate was calculated for the mean RTs of the dot-probe task. Afterwards, several 2 (group: clinical vs. control sample) × 2 (peer victimization: high vs. low) analyses of covariance (ANCOVAs) were conducted to examine the different hypothesized attentional biases dependent on the extent of diagnostic status and peer victimization. To date, the FBS lacks a representative norm sample and validated cutoff scores for peer victimization. In line with previous studies [81, 91, 92], therefore, a median-split of the FBS was used to categorize the samples into high vs low peer victimized participants. To be included in the high peer victimization group, participants had to score higher than the median (FBS total > 11; *n* = 28) on the FBS [89]. Participants scoring lower than that were assigned to the low peer victimization group (*n* = 33). Next, for explorative reasons the existence of absolute attentional biases was examined by applying planned *t*-tests for each attentional bias index score. Specifically, the bias and index scores for all four combinations of group (clinical vs. control) and peer victimization (high vs. low) were compared to zero. Additionally, because the clinical sample and the healthy control sample differed significantly on some subscales of the CTQ, all ANOVAs were carried out as analyses of covariance (ANCOVAs) with age and the CTQ sum score serving as covariates to control for the influence of childhood maltreatment within the family. As the pattern of results did not change, only ANCOVAs with age serving as the covariate are reported. For the ANCOVAs, partial eta-squared (η^2^) values were reported to demonstrate the size of effects such that 0.01 represents a small effect, 0.06 a medium effect, and 0.14 a large effect [107]**.**

## Results

The average age of the total sample was *M* = 24.59 years (*SD* = 4.97). However, mean age differed significantly between the two samples (clinical sample: *M* = 26.20 years, *SD* = 6.12; control sample: *M* = 23.03 years, *SD* = 2.83; *t* (59) = 2.61; *p* = .012). Participants’ means on the assessments and diagnoses are presented in Table 1.

### Emotional Stroop

The initial repeated measures ANCOVA showed a significant interaction effect of Valence x Peer victimization, *F* (2, 104) = 3.30; *p* = .041; *η*^*2*^ = .060. Further main or interaction effects did not reach significance (all *p*’s > .05; see Table 4). Mean RTs and standard deviations are presented in Table 5. In a next step, the hypotheses that individuals diagnosed with a psychiatric disorder as well as peer victimized individuals show an attentional bias towards negative compared to neutral adjectives were tested. Additionally, the influence of diagnostic status and peer victimization on the processing of positive compared to neutral adjectives was analyzed.

**Table 4:** *F*, *p*, and *η2* values for all ANCOVAs of the Emotional Stroop Task

	*df*	*F*	*p*	*η* ^*2*^
Omnibus ANCOVA				
Age	1, 52	3.37	.072	.061
Valence	2, 104	.89	.413	.017
Group	1, 52	.06	.815	.001
Peer victimization	1, 52	.80	.376	.015
Valence x Group	2, 104	.32	.725	.006
Valence x Peer victimization	2, 104	3.30	.041*	.060
Group x Peer victimization	1, 52	1.38	.246	.026
Valence x Group x Peer victimization	2, 104	.90	.409	.017
Negative-neutral trials				
Age	1, 54	.34	.563	.006
Group	1, 54	.11	.747	.002
Peer victimization	1, 54	3.90	.053	.067
Group x Peer victimization	1, 54	.19	.662	.004
Positive-neutral trials				
Age	1, 52	2.46	.123	.045
Group	1, 52	.06	.803	.001
Peer victimization	1, 52	4.57	.037*	.081
Group x Peer victimization	1, 52	.37	.548	.007

* *p* < .05

**Table 5:** Mean RTs in milliseconds and standard deviations of the Emotional Stroop task (*N* = 59)

	Psychiatric patients		Healthy controls	
	High peer victimization (*n* = 20)	Low peer victimization (*n* = 9)	High peer victimization (*n* = 7)	Low peer victimization (*n* = 23)
Negative adjectives, *M* (*SD*)	4089.78 (821.11)	3546.85 (732.36)	3592.22 (654.41)	3693.30 (780.44)
Neutral adjectives, *M* (*SD*)	3964.37 (868.04)	3747.75 (1056.72)	3466.32 (393.99)	3739.61 (787.54)
Positive adjectives, *M* (*SD*)	4011.57 (765.06)	3627.74 (756.86)	3598.98 (310.69)	3598.83 (679.25)
Emotional Stroop Index (negative – neutral), *M* (*SD*)	112.75 (368.30)	− 149.25 (451.77)	125.91 (363.40)	−46.31 (283.22)
Emotional Stroop Index (positive – neutral), *M* (*SD*)	47.20 (314.76)	−120.01 (385.77)	132.66 (288.82)	−140.78 (263.91)

**Fig. 1:**
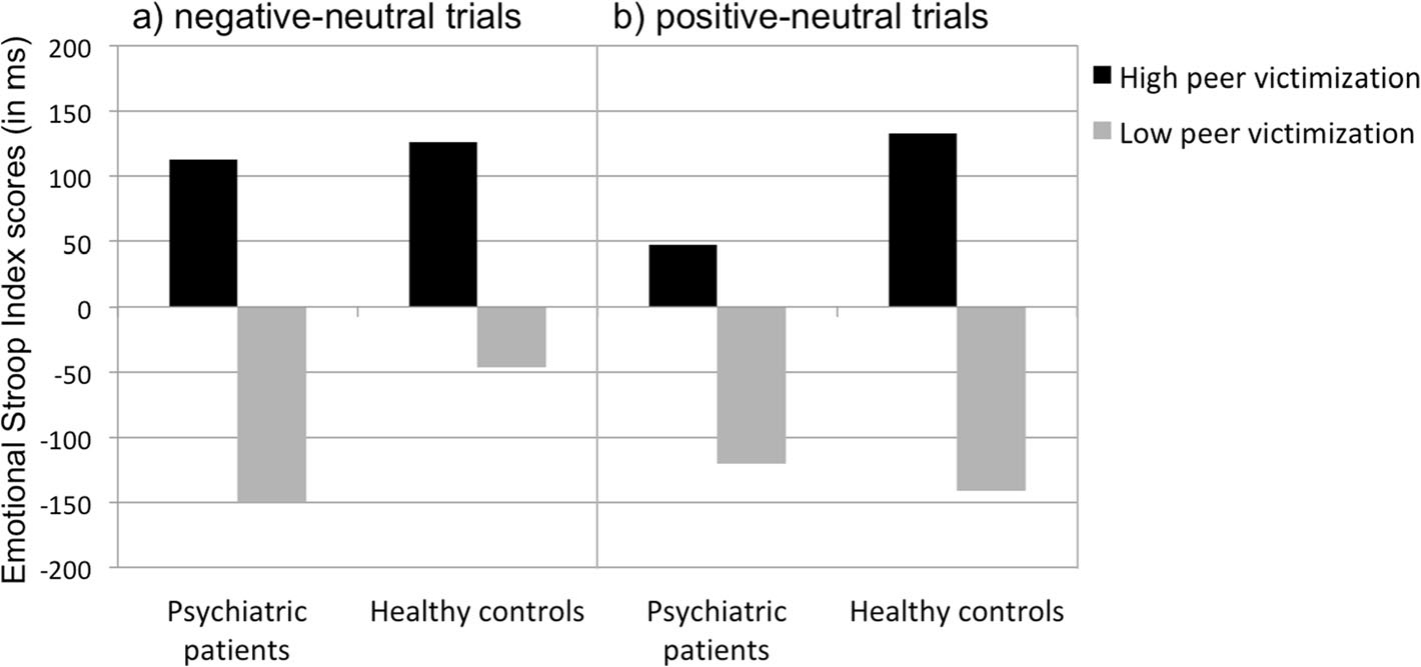
Emotional Stroop Index scores (in ms) of participants for **a**) negative-neutral trials, and **b**) positive-neutral trials

For negative-neutral trials, the age-corrected ANOVA revealed a marginally significant main effect of peer victimization, *F* (1, 54) = 3.90; *p* = .053; *η*^*2*^ = .067 (see Fig. 1). The main effect of group was not significant, *F* (1, 54) = .11; *p* = .747; *η*^*2*^ = .002. The interaction effect of Group x Peer victimization did also not show significant differences, *F* (1, 54) = .19; *p* = .662; *η*^*2*^ = .004.

For positive-neutral trials, the age-corrected ANOVA showed a significant main effect of peer victimization, *F* (1, 52) = 4.57; *p* = .037; *η*^*2*^ = .081. There was no main effect of group, *F* (1, 52) = .06; *p =* .803; *η*^*2*^ = .001. Similarly, the interaction of Group x Peer victimization did not reach significance, *F* (1, 52) = .37; *p* = .548; *η*^*2*^ = .007. Additionally, the Emotional Stroop Index score for the positive-neutral trials showed a significant difference from zero in healthy controls without a history of peer victimization, *t* (22) = − 2.56; *p* = .018. In the explorative analyses, all other Emotional Stroop index scores did not differ significantly from zero (all *p*’s > .05).

### Dot-probe task

Initially, in the omnibus repeated measures ANCOVA significant main effects of valence and group were found (valence: *F* (1, 53) = 5.89; *p* = .019; *η*^*2*^ = .100; group: *F* (1, 53) = 16.21; *p <* .001; *η*^*2*^ = .234). Additionally, the interaction effects of Location x Group, *F* (1, 53) = 4.77; *p* = .033; *η*^*2*^ = .083, and Location x Peer victimization, *F* (1, 53) = 6.20; *p* = .016; *η*^*2*^ = .105, reached significance. Further significant main or interaction effects were not found (all *p*’s > .05; see Table 6). Mean RTs and standard deviations are presented in Table 7. Next, the hypotheses that individuals diagnosed with a psychiatric disorder as well as individuals reporting high levels of peer victimization show attentional biases towards negative compared to neutral adjectives were examined for the three attentional bias indeces of the dot-probe task. Similarly, attentional biases in positive versus neutral trials were explored.

**Table 6:** *F*, *p*, and *η2* values for all ANCOVAs of the Dot-Probe Task

	*df*	*F*	*p*	*η* ^*2*^
*Omnibus ANCOVA*				
Age	1, 53	.01	.943	< .001
Valence	1, 53	5.89	.019*	.100
Location of dot	1, 53	1.86	.178	.034
Group	1, 53	16.21	< .001***	.234
Peer victimization	1, 53	1.45	.233	.027
Valence x Location of dot	1, 53	.30	.584	.006
Valence x Group	1, 53	.92	.342	.017
Valence x Peer victimization	1, 53	.02	.888	< .001
Location of dot x Group	1, 53	4.77	.033*	.083
Location of dot x Peer victimization	1, 53	6.20	.016*	.105
Group x Peer victimization	1, 53	1.74	.193	.032
Location of dot x Group x Peer victimization	1, 53	.81	.372	.015
Valence x Group x Peer victimization	1, 53	.09	.767	.002
Valence x Location of dot x Group	1, 53	.01	.945	< .001
Valence x Location of dot x Peer victimization	1, 53	1.40	.243	.026
Valence x Location of dot x Group x Peer victimization	1, 53	.19	.666	.004
*Attentional Bias Score*				
*Negative-neutral trials*				
Age	1, 53	1.64	.198	.031
Group	1, 53	2.46	.123	.044
Peer victimization	1, 53	6.27	.015*	.106
Group x Peer victimization	1, 53	.83	.367	.015
*Positive-neutral trials*				
Age	1, 53	.49	.489	.009
Group	1, 53	2.66	.109	.048
Peer victimization	1, 53	1.14	.291	.021
Group x Peer victimization	1, 53	.15	.704	.003
*Orienting Index Score*				
*Negative-neutral trials*				
Age	1, 53	.01	.909	< .001
Group	1, 53	.63	.430	.012
Peer victimization	1, 53	2.10	.153	.038
Group x Peer victimization	1, 53	.80	.374	.015
*Positive-neutral trials*				
Age	1, 53	2.69	.107	.048
Group	1, 53	2.75	.103	.049
Peer victimization	1, 53	.08	.779	.002
Group x Peer victimization	1, 53	.07	.795	.001
*Disengaging Index Score*				
*Negative-neutral trials*				
Age	1, 53	2.92	.093	.052
Group	1, 53	1.49	.228	.027
Peer victimization	1, 53	3.20	.080	.057
Group x Peer victimization	1, 53	.10	.751	.002
*Positive-neutral trials*				
Age	1, 53	.43	.514	.008
Group	1, 53	.20	.660	.004
Peer victimization	1, 53	1.04	.313	.019
Group x Peer victimization	1, 53	.05	.828	.001

* *p* < .05, ** *p* < .01, *** *p* < .001

**Table 7:** Mean RTs in milliseconds and standard deviations of the dot-probe task (*N* = 58)

	Psychiatric patients		Healthy controls	
	High peer victimization (*n* = 20)	Low peer victimization (*n* = 9)	High peer victimization (*n* = 7)	Low peer victimization (*n* = 22)
Negative-neutral pairs				
Negative location, *M* (*SD*)	1036.03 (141.69)	941.75 (121.70)	868.32 (55.38)	863.45 (60.34)
Neutral location, *M* (*SD*)	1027.34 (134.29)	963.08 (120.66)	856.66 (51.52)	865.62 (61.39)
Positive-neutral pairs				
Positive location, *M* (*SD*)	1027.89 (133.89)	948.96 (116.65)	866.30 (65.14)	866.82 (66.10)
Neutral location, *M* (*SD*)	1027.97 (128.97)	960.30 (113.08)	856.69 (54.38)	862.42 (59.93)
Neutral-neutral pairs				
Neutral location, *M* (*SD*)	1031.93 (134.69)	954.19 (117.58)	864.21 (51.79)	863.42 (60.28)
Attentional Bias Score (negative – neutral), *M* (*SD*)	−8.69 (40.46)	21.33 (36.90)	−11.66 (20.94)	2.16 (23.68)
Attentional Bias Score (positive – neutral), *M* (*SD*)	.08 (35.63)	11.34 (33.02)	−9.61 (27.94)	−4.39 (19.12)
Orienting Index Score (negative – neutral), *M* (*SD*)	−4.10 (28.76)	12.43 (21.01)	−4.11 (19.36)	−.03 (18.79)
Orienting Index Score (positive – neutral), *M* (*SD*)	4.05 (27.03)	5.22 (20.94)	−2.09 (21.96)	−3.39 (18.19)
Disengaging Index Score (negative – neutral), *M* (*SD*)	−4.59 (36.80)	8.90 (21.89)	−7.55 (15.03)	2.19 (18.31)
Disengaging Index Score (positive – neutral), *M* (*SD*)	−3.97 (27.13)	6.11 (28.60)	−7.52 (19.29)	−1.00 (20.57)

### Attentional Bias score

**Fig. 2:**
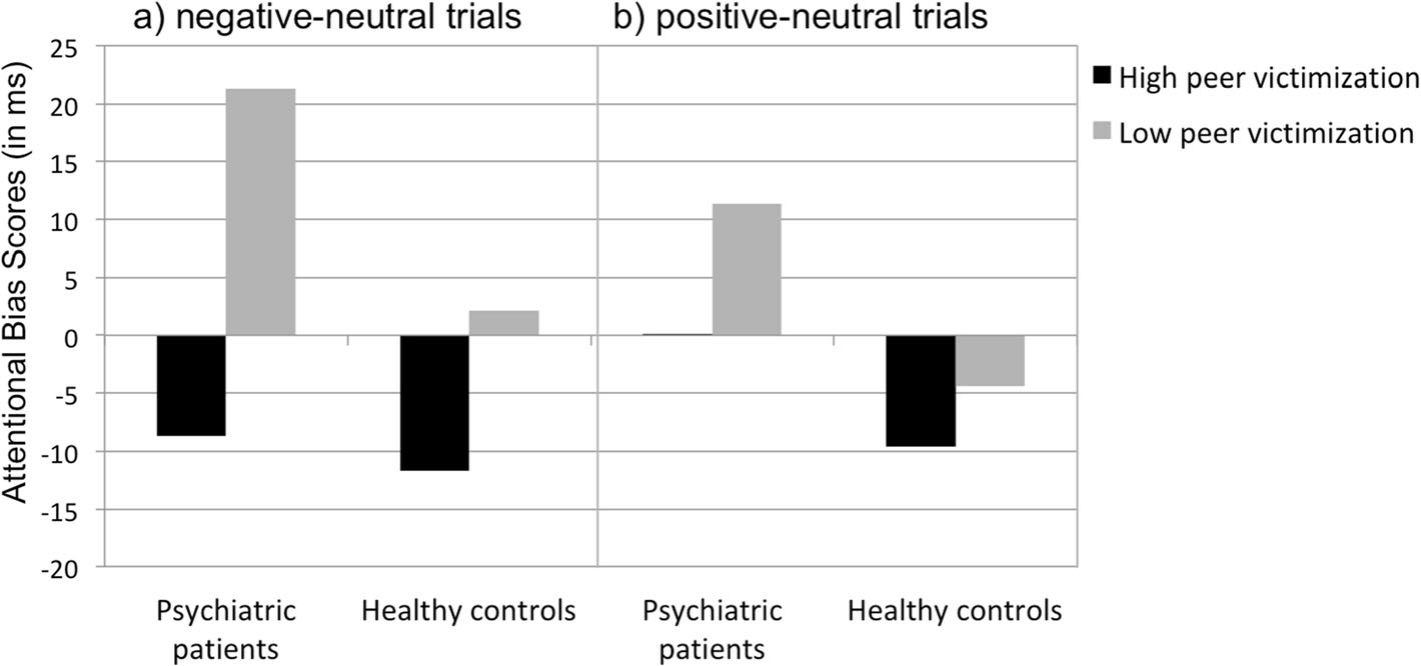
Attentional Bias Scores (in ms) of participants for **a**) negative-neutral trials, and **b**) positive-neutral trials in the dot-probe task

The ANOVA with age serving as a covariate revealed a significant main effect for peer victimization, *F* (1, 53) = 6.27; *p* = .015; *η*^*2*^ = .106, for the general attentional bias score when comparing negative to neutral words (see Fig. 2). Here, the main effect of group, *F* (1, 53) = 2.46; *p* = .123; *η*^*2*^ = .044, and the interaction effect of Group x Peer victimization, *F* (1, 53) = .83; *p* = .367; *η*^*2*^ = .015, were not found to be significant. The age-corrected ANOVA for the attentional bias score for positive compared to neutral words did not show any significant effects (all *p*’s > .05; see Table 6). Similarly, no absolute attentional bias scores differed significantly from zero in the explorative analyses (all *p*’s > .05) indicating that only relative attentional biases were present.

### Orienting index score

For the orienting index score comparing negative to neutral words, the age-corrected ANOVA did not reveal any significant effects (all *p*’s > .05; see Table 6). For the orienting index score examining positive and neutral words, the ANOVA showed similar results. Here, no significant effects were found (all *p*’s > .05; see Table 6). Again, there were no significant absolute bias scores in the explorative analyses (all *p*’s > .05).

### Disengaging index score

The ANOVAs with age serving as the covariate for the analyses of the effects of peer victimization and group on the difficulty to disengage from negative or positive words showed no significant effects (all *p*’s > .05; see Table 6). However, the main effect of peer victimization indicated a tendency towards significance when comparing negative to neutral words, *F* (1, 53) = 3.20; *p* = .080; *η*^*2*^ = .057. In the explorative analyses, the analyses of absolute bias scores showed no significant effects (all *p*’s > .05).

## Discussion

In the current study two tasks measuring attentional biases were administered. Both tasks showed that attentional processes were linked to a higher degree to the experience of peer victimization in childhood and adolescence than to the current diagnostic status of the participants. As a function of earlier peer victimization, participants’ responses to negative as well as positive words compared to neutral words shifted from faster to delayed reactions. While individuals reporting low levels of peer victimization were faster in the color-naming of emotionally valenced compared to neutral words, color-naming of negative and positive words was interfered (i.e., slower) in highly peer victimized participants. With respect to the results of the dot-probe task, peer victimized participants’ responding can be interpreted as attentional avoidance of emotional stimuli. Reported effect sizes of the significant effects were medium.

In accordance with studies indicating attentional biases in subjects with ACEs [56–61] and our hypotheses, peer victimized subjects showed delayed responses to color-naming negative compared to neutral adjectives in the Emotional Stroop task. However, in contrast to studies that reported facilitated attention towards [58, 60, 61] or difficulties to disengage from threatening stimuli in maltreated subjects [59], the dot-probe task utilized in the present study revealed that participants with a history of peer victimization avoided negative adjectives rather than detecting them faster or allocating their attention towards negative words. This is in accordance with findings of threat avoidance in maltreated children with PTSD [108]. Furthermore, Fani and colleagues (2011) reported that childhood maltreatment predicts attention bias scores beyond the effects of traumatic experiences in adulthood in a sample of PTSD patients [57]. Here, victims of childhood abuse showed an attentional bias towards happy relative to neutral faces, and reported to have experienced more PTSD avoidance and numbing symptoms. Since the attentional bias was not linked to other PTSD symptomatology, the authors concluded that the attentional bias may reflect avoidant tendencies rather than hyperattention to positive cues in this study [57]. As an explanation, it was suggested that maltreated subjects may have learned to selectively allocate their attention away from potential stressors as a means of coping with constant adversity. With respect to recent studies linking attentional avoidance to emotional regulation strategies [1, 31, 109–111], our results indicate that maltreated subjects may attempt to strategically regulate negative affect via distraction which may be due to an inavailability of other cognitive coping resources [2, 4, 112].

It may be speculated that for subjects encountering situations of peer abuse there are initially no active coping or behavioral resources based on a fight or flight stress response which would be associated with facilitated attention towards threat. Instead, subjects experiencing peer victimization may undergo a down-regulation of behavioral and attentional processes which are reflected by avoidance of threatening stimuli. This reasoning is supported by the fact that peer victimization is associated with blunted responses to stress [63–66, 68, 113–118]. Accordingly, attentional avoidance was linked to prefrontal cortex functioning which is also involved in the processing of social stress [1, 119–121]. Here, several studies showed an activation of prefrontal cortex areas, in particular the dorsal anterior cingulated cortex and the right ventrolateral prefrontal cortex [119–130], when subjects were socially excluded, which is a part of the wide range of experiences of peer victimization. Similarly, it has been suggested that attentional avoidance is related to strategic cognitive-regulatory processes that are also linked with higher-order cortical structures like the prefrontal cortex [1]. Hence, it may be assumed that negative words used in the present study were able to elicit a connotation to social stress experiences in peer victimized subjects. As a consequence, cognitive emotion regulation processes in higher-order cortical structures may have led to attentional avoidance of negative versus neutral words in subjects with a history of peer victimization. In accordance with this assumption, a recent study indicated higher neural activity in brain regions that are involved in social cognition and cognitive control in chronically victimized girls [131]. Effect sizes in our sample that was not explicitly recruited with respect to high amounts of peer victimization experiences were already found to be medium. It is likely that attentional biases may even be larger in samples consisting of individuals that were screened for chronical victimization.

However, since the pattern of results did not differ between negative and positive adjectives, the present study indicated a general emotion-avoidant, rather than threat-avoidant, attentional style in subjects with a history of peer victimization. This finding is in accordance with other reports of attentional biases in reaction to different emotional stimuli [132–134] as well as with generalized hyper-sensitive and hyper-vigilant reactions towards emotional stimuli in matreated individuals [116, 135–137]. Albeit, some of the referenced studies deal with methodological shortcomings (e.g., balancing of word frequency [133]) which should be addressed in future studies.

Recently, Rudolph, Troop-Gordon, and Granger (2010) reported that anticipatory cortisol and salivary alpha amylase activation was increased in victimized children who were informed that they would be interacting with unfamiliar peers [138]. It was suggested that enhanced physiological activation in victimized subjects reflects a hyper-alertness to social threat [138]. Accordingly, the present result patterns in subjects with a history of peer victimization may also illustrate a hyper-alertness or hyper-vigilance to social threat. That is, based on negative social experiences victimized subjects may be more likely to anticipate social threat and negative consequences for their well-being even when confronted with positive stimuli. In a next step, this anticipation of social threat and its consequences may lead to hyper-sensitive emotion regulation processes so that attentional avoidance reactions are generalized from negative to all kinds of emotional stimuli. In agreement with this, children who reported adverse experiences have been found to be more likely to show vigilance and cognitive sensitivity to social threat [138–140]. Moreover, this hyper-alterness may represent the link between peer victimization and the development of psychopathology documented in several studies [71, 73–75, 79, 81].

Contrasting with a wealth of research [2, 16, 27, 29, 42] and our hypotheses, the present study did not find a significant influence of psychopathology or the clinical diagnostic status on attentional biases. However, on a descriptive level, subjects of the clinical sample, overall, showed a facilitated attentional orienting towards negative words. Interestingly, in dependence of experiences of peer victimization, the quality of the attentional bias, rather than quantity, depicted in the present study changes. While subjects scoring low in peer victimization showed an allocation of attention towards negative stimuli, attentional allocation shifted into avoidance of threatening stimuli in highly victimized subjects. Hence, it may be concluded that attentional biases are linked to psychopathology, but the quality (i.e., the allocation of attention) of these biases is determined by further factors as early life experiences (i.e., peer victimization).

Addressing the fact that diagnoses in the present study were rather heterogeneous and therefore may be accompanied by a varying magnitude and direction of biases, this conclusion also refers to the finding that peer victimization influenced attentional processes even beyond the effects of trait anxiety. However, most studies indicating attentional biases in psychiatric disorders, and particularly anxiety disorders, used stimuli that were either threat-, fear- or disorder-related [2, 16, 49]. The present study, however, did not use stimuli that were related to a certain disorder or subject of fear, but negative, neutral, and positive adjectives that may have provided a social evaluation connotation. It may be speculated that the present stimuli may rather be related to experiences of peer victimization than to psychopathology. Hence, the null effects found for the influence of psychopathology on attentional biases may be due to the utilized stimuli set. Accordingly, adjectives that reflect depressive or anxious cognitions and experiences (e.g., sad, afraid, nervous, worried) may rather have been suitable to elicit attentional biases in the clinical sample. Consequently, future studies should use a set of stimuli that includes both peer victimization related social evaluative adjectives as well as disorder- or fear-related adjectives to disentangle different effects of victimization and psychopathology on the processing of emotional words.

Moreover, the present study has several additional limitations. The assessment of peer victimization was based on self-report and retrospective accounts and may be subjected to recall biases [141]. Analyses of the validity of retrospective reports, however, suggest that these biases are not large enough to invalidate retrospective studies [142]. In addition, under-reporting of child maltreatment was more prevalent than over-reporting in retrospective assessments. Moreover, the set of stimuli that was used in the present study may not have been arousing or threatening enough to generate attentional biases. According to Mogg and Bradley (1998), different attention to threat in subjects with varying levels of trait anxiety depends on the valence of stimuli [4]. Here, the use of pictures rather than words may have been useful to elicit higher levels of arousal and potentially more elevated attentional biases [143]. Furthermore, stimuli were not masked in their presentation. Therefore, underlying mechanisms and stage of processing (automatic versus strategic) could not be examined systematically. The generalizability of our findings is limited. Affective disorders comprised about 60% of the psychiatric patients sample while anxiety disorders were rather under-represented. This may also be reflected by the trait anxiety scores which did not differ from the control group. With respect to the literature on attentional biases in anxiety disorders [15], it may be assumed that a higher amount of patients with anxiety disorders may have resulted in greater attentional biases in the psychiatric sample. Accordingly, considering the diagnostic status as a single, categorical variable may limit the validity of the current study as it may conceal differences in attention processes in various psychiatric disorders. Future studies should address this shortcoming by recruiting psychiatric samples comprising sufficiently largh enough sub-samples of different psychiatric disorders to account for disorder-related differences in emotion processing. Additionally, the current study is limited by the small sample size and sample composition. With a total of 61 participants, the analyses may have been underpowered to reveal potential effects. Next, the clinical sample and the healthy control sample differed in age which had to be controlled for in the analyses again weakening the power of analyses. Moreover, the sample was relatively young, with subjects who are predominantly single. Limitations in sample size and composition should be addressed in future studies using larger and more representative samples. Additionally, the present study is limited by the fact that information about the diagnostic status (i.e., number and type of diagnoses) of the clinical sample was obtained from the subjects’ health records and no clinical interviews were carried out in this sample. Therefore, reliability and validity of clinical diagnoses may be limited. Future studies should address this point by carrying out clinical interviews in all subjects.

## Conclusion

Recently, a growing body of literature has emphasized the role of peer victimization as a major public health concern. In school children, reports of repeated victimizations range from 10 to 20%, with periodic adversities being indicated even more frequently [138, 144, 145]. This prevalence rates of victimization become even more alarming with respect to reports indicating that the outcomes of emotional maltreatment are as harmful as the consequences of sexual and/or physical maltreatment [70, 72]. However, knowledge about the mechanisms linking peer victimization and psychopathology remains elusive. Here, the effects of ACEs on emotion processing styles may play a crucial role [57, 146]. With respect to the results of the present study, peer victimization in and of itself is associated with biases in emotion processing. Hence, biased emotional processing styles may be a mechanism that link peer victimization to a wide range of latter psychopathology. In this conceptualization, it may be assumed that individuals experiencing peer victimization in their childhood and adolescence are more likely to develop an avoidant attentional and emotional processing style. If this attentional bias persists during development, it may enhance an inappropriate processing of relevant environmental emotional information. As a consequence, peer victimized subjects may be more vulnerable to the development of psychopathology [71, 73–81]. This effect may be even bigger if individuals have experienced multiple adverse events [57, 146]. Hence, a better understanding of the specific characteristics in the processing of emotional and neutral stimuli in the wake of peer victimisation could help to address short and long term consequences for victims. For instance, treatment of peer victimization related psychiatric disorders may implement cognitive modules targeting attention. Accordingly, attentional bias modification has been proposed to be the first of a two-step treatment approach for people at risk for developing psychiatric disorders [15]. Here, victims of peer victimization would run an attentional bias modification training first before traditional cognitive behavioral therapy is offered. However, future studies on the efficacy of attentional bias modification in peer victimized individuals and its consequences on psychopathology and other negative outcomes are needed. Lastly, the current study provided evidence that experiences of childhood emotional maltreatment are associated with attentional biases to emotionally stimuli in adulthood. Therefore, the implementation of measures of childhood maltreatment in future studies on attentional biases in clinical as well as healthy samples is strongly suggested.

## Abbreviations

ACEs: adverse child experiences
BDI-II: Beck Depression Inventory II
BSI: Brief Symptom Inventory
CTQ: Childhood Trauma Questionnaire
FBS: *Fragebogen zu belastenden Sozialerfahrungen* [Adverse Social Experiences Questionnaire]
GAD: generalized anxiety disorder
M.I.N.I.: Mini International Neuropsychiatric Interview
OCD: obsessive-compulsive disorder
PTSD: posttraumatic stress disorder
RT: reaction times
SAD: social anxiety disorder
